# Diseases of the Respiratory System

**Published:** 1904-10-15

**Authors:** 


### DISEASES OF THE RESPIRATORY SYSTEM.

*(Concluded from page 29.)*

**Garlic Treatment.**—This substance appears to have been independently advocated by Cavazzani in Italy, Poore in England, and Minchin in Ireland.[5] The latter has employed it for over five years and reports good results, especially in very early and in chronic fibroid cases. He recommends that prolonged inhalations should be given, the juice being placed on the sponge of an ordinary metal inhaler. It may also be given internally, or the pulped garlic may be applied as a poultice to the affected area. It has, however, a variable tendency to blister the skin. It may be applied to the broken or unbroken skin, but should never be injected into joints, as it is a violent irritant. Minchin has had excellent results with garlic poultices as a local application in lupus.

**Open-air Treatment.**—The effects of sea voyages and residence in sanatoria or at high altitudes are explained by McLaughlin[6] as follows:—Owing to natural partial immunity pure tuberculous infections in man become walled-off by fibrous tissue. No intoxication takes place, and recovery ensues. When, however, pus cocci are added to the infection, ulceration takes place in the lesions, and a latent tuberculosis becomes active. All bacteria under cultivation tend to lose their toxic properties, and eventually to die out. If, therefore, a patient with mixed infection is made to breathe sterile air, and his surroundings are kept aseptic, the pus cocci gradually become non-virulent and die out, and the organism deals with the pure tuberculous affection in the usual way. When, however, the patient returns to ordinary surroundings reinfection may occur, and the latent phthisis become again ulcerative and active.

**Tuberculosis and other Diseases.**—In a review of various diseases occasionally found in conjunction with pulmonary tuberculosis, Parkes Weber[7] comes to the conclusion that as regards the heart, congenital pulmonary stenosis and heart diseases tending to anæmia of the lungs produce a susceptibility to phthisis, while those producing engorgement hinder it. Tuberculosis leads to fatty infiltration, and occasionally amyloid degeneration of the liver, while in some cases cirrhosis may be set up, though probably the frequent association of tuberculosis and cirrhosis may be due to a common cause, namely alcoholism. This is an important factor in predisposing to tuberculosis, as it impairs metabolism and favours tissue degeneration; a similar relation obtains between alcoholism and other infective diseases. Moreover, the children of alcoholics often show weakened powers of resistance. The kidneys are often affected, either by slight amyloid changes, actual tuberculosis, or mixed nephritis due to toxin elimination. The antagonism between gout and tuberculosis which is an actual fact may be due to the large meat-eating proclivities which induced the gout. Hence the value of full meat diet in phthisis. The antagonism between cancer and tuberculosis if it exists may be due to the varied age incidence of the two diseases or to the fact that cancer often attacks robust people who live well. Cancerous cachexia may predispose to phthisis. Lymphadenoma certainly bears a close relation to tuberculosis, but in leuchæmia, though the diseases are sometimes associated, a retrogression of the former sometimes takes place when tubercle supervenes. Phthisis is frequently a complication of diabetes—the sugar in the tissues possibly aiding the growth of organisms. Its disappearance with the onset of tuberculosis may be due to the action of bacterial toxins. The position of syphilis seems doubtful, some holding that it antagonises tuberculosis to some extent. Though alcoholism certainly predisposes to both tuberculosis and peripheral neuritis, the latter may not necessarily arise from alcoholism alone, and in some cases is probably due to tuberculous or other bacterial toxins.

[5] Lancet, Feb. 13, 1904, p. 481. [6] Med. Rec., Mar. 5, 1904 p. 367. [7] Lancet, April 2, 1904, p. 924.

